# Effects of Different Heating and Cooling Rates during Solution Treatment on Microstructure and Properties of AA7050 Alloy Wires

**DOI:** 10.3390/ma17020310

**Published:** 2024-01-08

**Authors:** Xinyu Gao, Guanjun Gao, Zhihui Li, Xiwu Li, Lizhen Yan, Yongan Zhang, Baiqing Xiong

**Affiliations:** 1 State Key Laboratory of Non-Ferrous Metals and Processes, China GRINM Group Co., Ltd., Beijing 100088, China; god_xinyu@163.com (X.G.); lixiwu2020@126.com (X.L.); yanlizhen@grinm.com (L.Y.); zhangyongan@grinm.com (Y.Z.); xiongbq@grinm.com (B.X.); 2 Northeast Light Alloy Co., Ltd., Harbin 150060, China; 3 General Research Institute for Nonferrous Metals, Beijing 100088, China; 4 GRIMAT Engineering Institute Co., Ltd., Beijing 101407, China

**Keywords:** AA7050 alloy wires, solution treatment, TEM analysis, precipitates behavior, natural aging

## Abstract

In the present study, the effects of varying heating and cooling rates during the solution treatment process on the microstructure and properties of AA7050 alloy wires were investigated using tensile tests, metallographic microscopy, electron backscattered diffraction, and transmission electron microscopy. It was found that the recrystallized grain size of the alloy, subjected to method of rapid heating, exhibited a smaller and more uniform distribution in comparison to method of slow heating. The low density of η′ strengthening phases after the artificial aging treatment was formed using air cooling method. Meanwhile, by using the water quenching method sufficient solute atoms and more nucleation sites were provided resulting in a large number of η′ strengthening phases being formed. In addition, the alloy processed using the water quenching method displayed higher strength than that treated using the air cooling method for the T6 and T73 states. Furthermore, coarse precipitates formed and less clusters were observed in the matrix, while high density nanoscale clusters and no continuous precipitation are formed when using the water quenching method.

## 1. Introduction

Aircraft aluminum alloy structural materials used in major components such as the fuselage, wings, landing gear, and empennage are subject to a range of loading/forces [1,2,3,4,5,6,7]. The assembly of these components involves the use of thousands of fasteners [8,9,10,11,12]. Addressing the imperative of weight reduction in the aircraft industry, AA7050 aluminum alloy wire, belonging to the Al–Zn–Mg–(Cu) series, is employed. This alloy is well-suited for aluminum fastener applications, given its commendable mechanical strength and workability. Notably, the alloy exhibits enhanced fracture toughness and corrosion resistance, attributable to its elevated copper content and a marginally higher Zn/Mg ratio [13,14,15].

It is generally believed that the comprehensive properties of alloys are significantly influenced by the rates of heating and cooling during the solution treatment process [16,17,18,19]. At present, the effects of different heating and cooling rates during solution treatment on the microstructure and properties of alloys were studied. Wang et al. [16] observed the formation of more uniformly distributed equiaxed grains in the Al–Mg–Si alloy under rapid heating conditions. The recrystallization microstructure predominantly consisted of equiaxed grains when subjected to a swift heating rate, whereas a slow heating rate resulted in the development of elongated grains. Dumont et al. [18] conducted a comprehensive examination on the effect of the cooling rates during various quenching processes on the precipitation behaviors induced by quenching and the mechanical properties of the 7040 alloy. Additionally, Zhang et al. [19] reported findings on the influence of varying cooling rates on the inhomogeneous precipitation behaviors of 7021 and 7085 alloys through Jominy quenching tests. The focus of the aforementioned investigations is directed towards aluminum alloy extruded profiles or plates. Nevertheless, the effects of different heating and cooling rates during solution treatment on microstructure and properties of AA7050 alloy wire remain relatively limited.

In the practical industrial preparation process, variations exist in the heating and cooling rates among AA7050 alloy wires with distinct diameters during the solution treatment. In the present work, different heating rates matching the cooling rates of AA7050 alloy wires were employed during the solution treatment, thereby replicating the authentic heat treatment scenarios encountered in alloy wires of varying diameters. The objective is to provide technical insights supporting the effective implementation of the solution treatment in the industrial production of AA7050 alloy wires.

## 2. Materials and Methods

**Figure 1:**
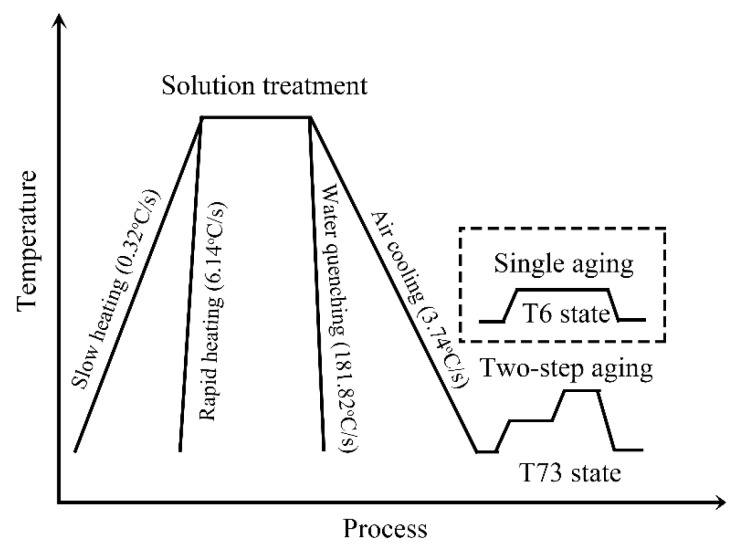
Schematic representation of the heat treatment procedure and the measured heating and cooling rates curves.

Typical AA7050 alloy wires with a diameter of 6.0 mm (H13 state) were prepared via casting, homogenization, hot rolling, intermediate annealing, and cold drawing. The chemical composition of the alloy used in this study was Al—6.18%, Zn—2.09%, Mg—2.17%, Cu—0.10%, Fe—0.11%, and Zr (wt.%). The wire samples, cut into 300 mm length, were given a solution heat treatment of 60 min at 475 °C in an air-circulation furnace using different heating rates. Using different cooling rates, the wire samples were cooled to room temperature (RT). One set of wire samples, with different heating and cooling rate solution treatments, was subjected to single-stage an artificial aging treatment of 24 h at 120 °C (T6 state). The other set of wire samples was first pre-aged at 120 °C for 7 h and then subjected to an aging treatment of 11 h at 175 °C (T73 state). The heat treatment methods of the samples with different heating and cooling rates are shown in the Table 1. A schematic representation of the heat treatment procedure and the measured heating and cooling rates are presented in Figure 1.

With a load speed of 3 mm/min, the tensile test was performed in accordance with the China Standard (GB/T 228-2002) [20] at RT using an MTS-WD3100 electronic universal testing machine (Instron Corporation, Norwood, MA, USA). The recrystallized grain size in the samples with different heating and cooling rates during solution treatment was studied using a JSM-7900F scanning electron microscope (SEM) (ZEISS Company, Oberkochen, Germany) equipped with electron backscatter diffraction (EBSD). The samples were prepared via mechanical grinding and electrolytic polishing. The scanning step size was 1.5 µm and the voltage was 20 kV. EBSD observations were acquired using OIM imaging software (AMETEK Inc., Berwyn, PA, USA). A Talos F200X G2 transmission electron microscope (TEM) (FEI Company, Hillsboro, OR, USA) was used to observe the precipitates in the wire samples after artificial aging treatment. The thin foils required for TEM were punched into 3 mm disks and then prepared in the Struers TenuPol-5 twin-jet electro polishing unit(Struers Company, USA) sing a solution of 30% HNO_3_ in methanol below −25 °C at an operating voltage of 18 V. All the TEM observations were performed along the <001>_Al_ zone axis.

## 3. Results

### 3.1. Mechanical Properties

**Figure 2:**
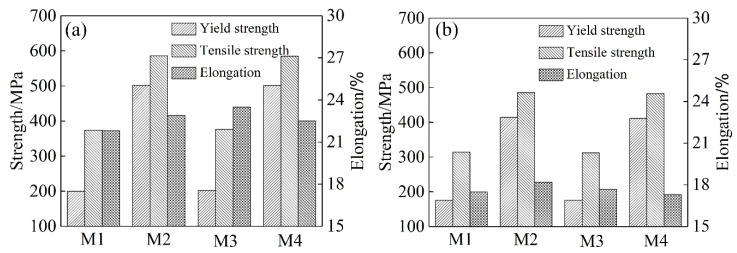
Mechanical properties of the wire samples with different solution treatments: (**a**) T6 state; (**b**) T73 state.

Figure 2 shows T6 and T73 state mechanical properties of the wire samples subjected to different solution treatments. As can be seen from Figure 2, the M1 and M3 samples of state T6 reached the similar strength level. The yield strength and tensile strength were ~200 MPa and ~375 MPa, respectively. The elongation was maintained at ~22.0%. Conversely, the M2 and M4 samples state T6 exhibit increased strength, with yield strength and tensile strength reaching ~500 MPa and ~585 MPa, respectively. While the elongation was not decreased, it was still kept at ~22.0%. The strength and elongation of the wire samples of T73 state decreased. For the M1 and M3 samples, the yield strength and tensile strength were decreased to ~175 MPa and ~310 MPa, respectively. The elongation reached ~17.5%. The yield strength and tensile strength of the M2 and M4 samples with T73 state decreased to ~410 MPa and ~485 MPa, respectively, accompanied by an elongation of ~17.5%.

### 3.2. Recrystallized Grains

**Figure 3:**
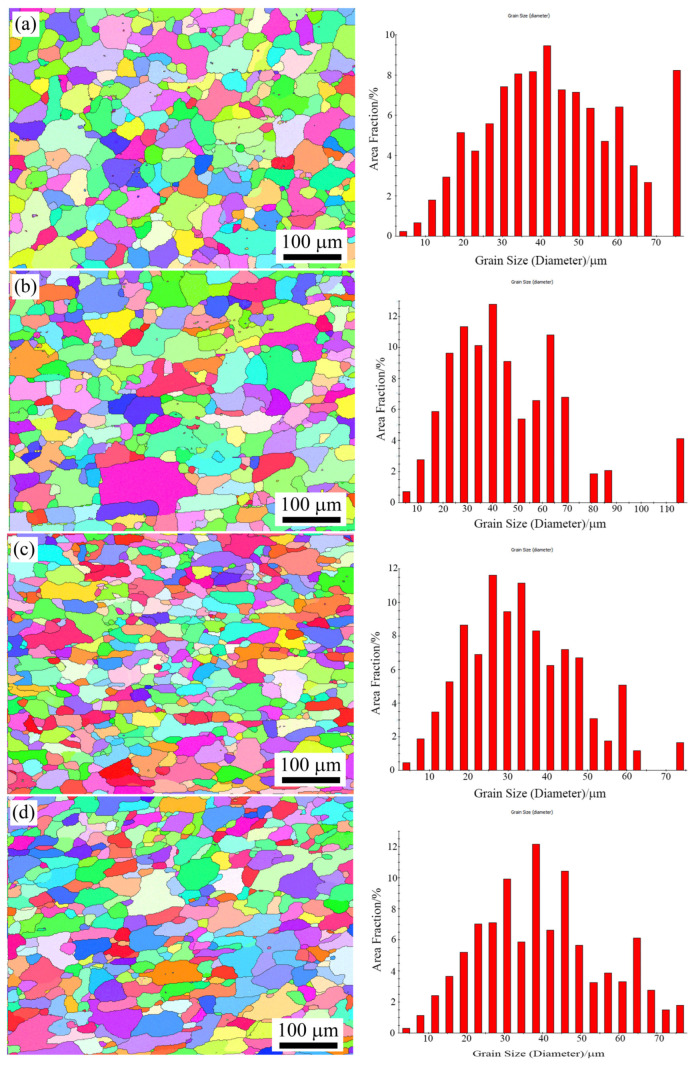
Recrystallized grains of the wire samples under different solution treatments: (**a**) M1; (**b**) M2; (**c**) M3; (**d**) M4.

Figure 3 shows recrystallized grains of the wire samples (lengthwise section) with different solution treatments. The wire samples were completely recrystallized after solution treatments. The average grain sizes of recrystallized grains were 43.1 μm for M1 and 45.7 μm for M2 samples. The grain size distribution exhibited non-uniformity, with some grains displaying abnormal coarseness. For the M1 and M2 samples, approximately 10% of the grains surpassed a size of 70 μm. On the contrary, the M3 and M4 samples demonstrated a more uniform grain size distribution. No abnormal grain growth occurred during solution treatments. Moreover, the average grain size of M3 and M4 samples were smaller, measuring 39.7 μm and 33.5 μm, respectively.

### 3.3. Intragranular and Grain Boundary Precipitation

**Figure 4:**
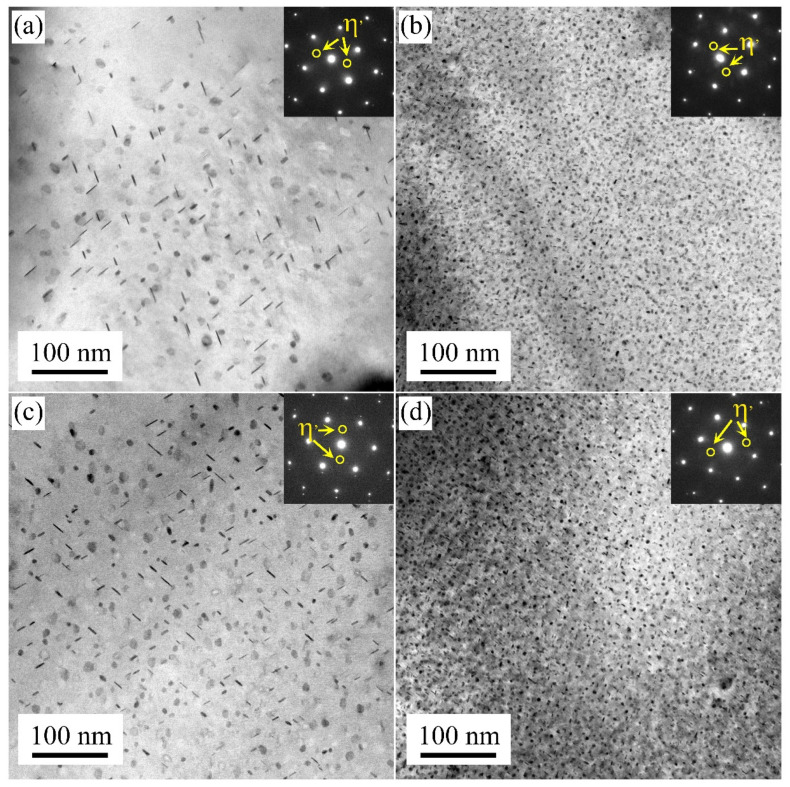
TEM bright field images of the M1 (**a**), M2 (**b**), M3 (**c**), and M4 (**d**) samples in their peak aging state (T6 state).

Figure 4 presents the TEM images of the samples at their peak aging state. Under the single-stage artificial aging treatment of 24 h at 120 °C, larger disc-shaped precipitates were observed in the M1 and M3 samples along the Al[110] axis. Diffraction spots at the 1/3 and 2/3{220} Al positions in the Al[110] axis were associated with η′ precipitates. The distribution of these precipitates was sparse. On the contrary, the strengthening precipitates formed in the M2 and M4 samples were denser than that of the M1 and M3 samples. The size of the precipitates was smaller. It was demonstrated that strengthening precipitates in water-quenched samples were smaller and denser compared to those in air-cooled samples in the T6 state.

**Figure 5:**
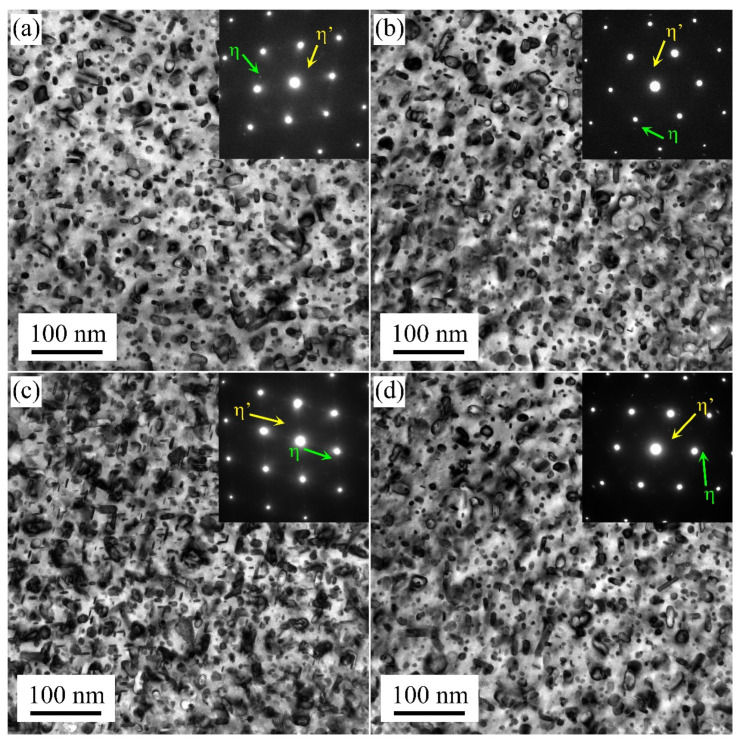
TEM bright field images of the M1 (**a**), M2 (**b**), M3 (**c**), and M4 (**d**) samples in their over-aged state (T73 state).

Figure 5 shows the TEM images of the samples under the over-aging state. With the first pre-aged at 120 °C for 7 h and second aged at 175 °C for 11 h treatment, the disc-shaped precipitates coarsened. Compared with the M2 and M4 samples, the difference in the precipitation size of the M1 and M3 samples reduced.

**Figure 6:**
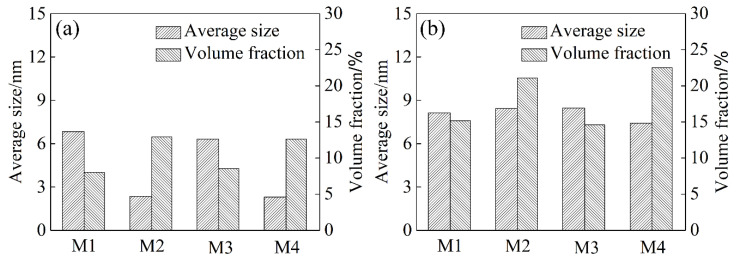
The average precipitation size and volume fraction of the M1, M2, M3 and M4 samples: (**a**) T6 state; (**b**) T73 state.

Figure 6 shows the average precipitation size and volume fraction of samples of the T6 and T73 states. Although the precipitation size of M1 and M3 samples exceeded that in the M2 and M4 samples, the volume fraction was lower. This was because the number density of the former was lower than that of the latter of the T6 state. In the T73 state, the precipitates coarsened, reaching a precipitation size of ~8 nm. Moreover, the precipitation volume fraction of samples increased accordingly. Nevertheless, the precipitation volume fraction of the M1 and M3 samples remained lower than that in M2 and M4 samples.

**Figure 7:**
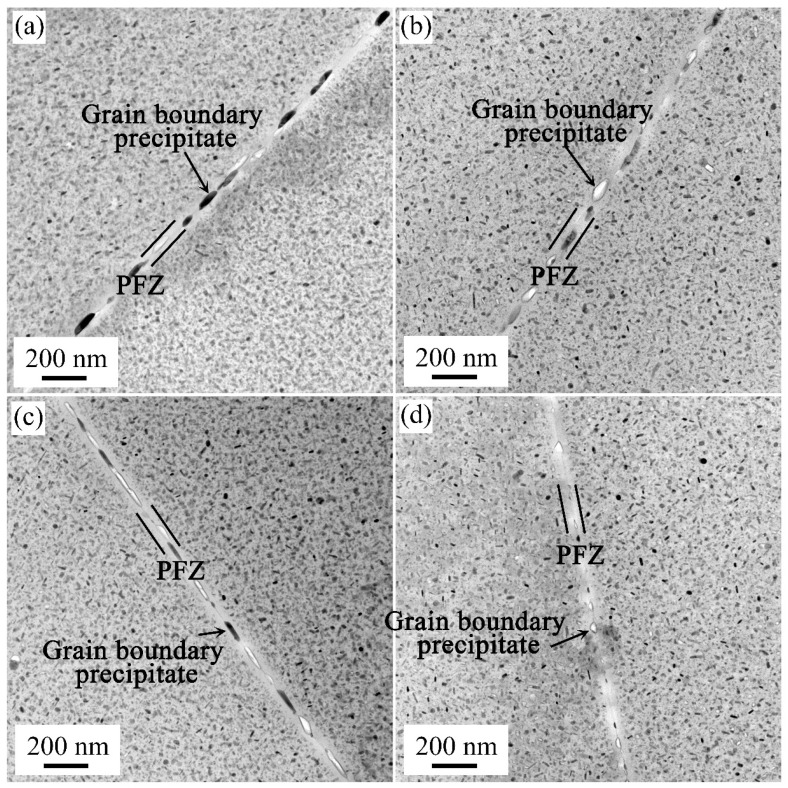
TEM images of grain boundaries of the M1 (**a**), M2 (**b**), M3 (**c**), and M4 (**d**) samples in the T73 state.

Figure 7 shows TEM images around grain boundaries of the samples in the T73 state. It was found that the intermittent precipitation was formed on grain boundaries. The grain boundary precipitation size was obviously larger than the inside grain in the T73 state. Besides, large widths of the PFZ (Precipitation-Free Zone) were observed in the grain boundaries. The formation of PFZ and grain boundary precipitates was strongly correlated.

### 3.4. Precipitation in Natural Aging State

**Figure 8:**
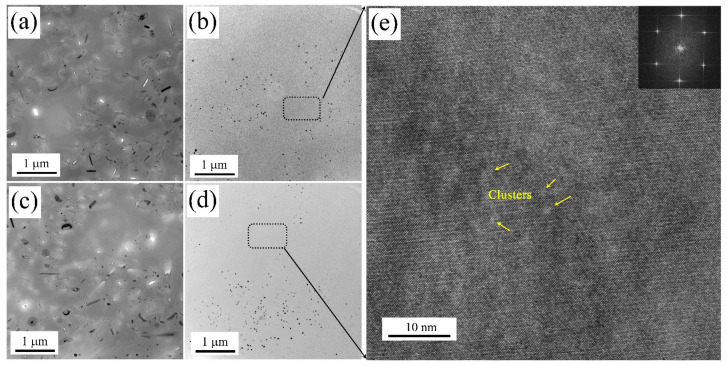
TEM images of the M1 (**a**), M2 (**b**), M3 (**c**), and M4 (**d**) samples in their natural aging state and the clusters of HRTEM images of the M2 and M4 samples (**e**).

Figure 8 shows the TEM and HRTEM images of samples in their natural aging state. In comparison with the M2 and M4 samples, larger precipitation was formed in the grains of the M1 and M3 samples, reaching the sub-micron scale. Conversely, only a limited amount of nano-scale precipitation was formed in the M2 and M4 samples. Figure 8e presents an HRTEM image of the matrix from the M2 and M4 samples, revealing a multitude of uniformly distributed nano-sized clusters formed during natural aging.

**Figure 9:**
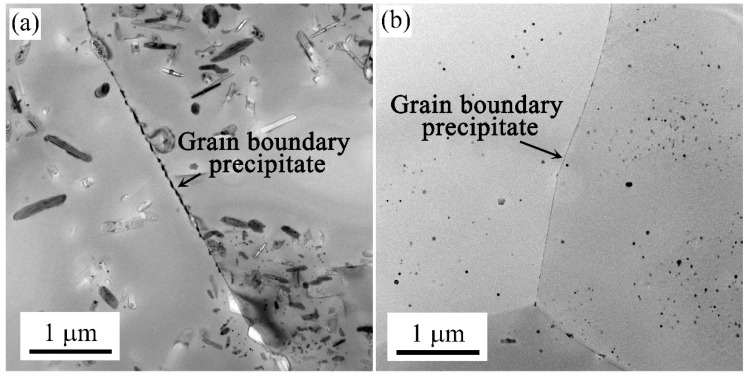
TEM images around grain boundaries of the M1 (**a**) and M2 (**b**) samples in their natural aging state.

Figure 9 shows TEM images of the grain boundaries of samples in their natural aging state. Coarse and continuous precipitation was formed on the grain boundary in the air-cooled samples (M1 and M3). Some of the precipitates at the grain boundaries fell off during TEM sample preparation. In contrast to the air-cooled samples, it was found that the formation of precipitation on grain boundaries can hardly be observed.

## 4. Discussion

The main difference in the samples between slow heating (M1 and M2) and rapid heating (M3 and M4) is grain size uniformity. The heating rate of solution treatment for the M1 and M2 samples was higher than that of the M3 and M4 samples, as shown in Figure 1. The latter samples exhibited more uniform and smaller grain sizes. A higher heating rate provides a greater driving force for recrystallization due to enhanced nucleation under elevated driving forces [21,22,23,24]. In addition, uniformity in heating also affects the recrystallization microstructure [25,26]. In cases of local overheating, recrystallized grain nuclei distribute unevenly in the matrix, resulting in coarser individual grains after solution treatment. Consequently, the recrystallized grain of the M1 and M2 samples was smaller and more homogeneous than those of the M3 and M4 samples, as shown in Figure 3.

The average precipitation size and volume fraction of alloys are important factors affecting the strength. Based on previous research, high-density η′ phases with the composition of Mg(Zn,Al,Cu)_2_ were the main strengthening precipitate in 7xxx alloys [27,28,29,30]. During the solution treatment, the soluble phase redissolved into the matrix. When the sample was cooled to RT using the air cooling method, the hold time at high temperature was prolonged. A large number of solute atoms and vacancies were consumed to form a coarsen η phase structure. This lowered the density of η′ strengthening phases after artificial aging treatment [28]. When the sample was rapidly quenched to RT (water quenching), there were many supersaturated solute atoms and vacancies in the matrix. During the subsequent artificial aging treatment, sufficient solute atoms and vacancies provided the raw materials and diffusion channels for the formation of η′ strengthening phases [28,30]. Additionally, with solute atoms and vacancies, more nucleation sites were provided for the formation of η′ phases. These factors collectively contribute to a noticeable increase in the strength and hardness of the sample after artificial aging treatment.

In the T6 state, the yield strength and tensile strength of M1 and M3 samples reached ~200 MPa and ~375 MPa, respectively. The elongation was maintained at ~22.0%. M2 and M4 samples increased to ~500 MPa and ~585 MPa, respectively. Meanwhile, the same level of elongation as M1 and M3 samples was maintained. With varying cooling rates during the solution treatment, the η′ strengthening phases volume fraction of M1 and M3 samples was lower than that of M2 and M4 samples. High-density precipitation imposes greater resistance to dislocation motion. It has the strongest pinning force on a moving dislocation and, therefore, they are most effective in strengthening, leading to the high-strength of the alloy [31,32]. Hence, the strength of the M2 and M4 samples with T6 state surpassed that of the M1 and M3 samples.

In the T73 state, the strength of the wire samples decreased compared to the T6 state. For the M1 and M3 samples, the yield strength and tensile strength were decreased to ~175 MPa and ~310 MPa, respectively. The yield strength and tensile strength of the M2 and M4 samples in the T73 state decreased to ~410 MPa and ~485 MPa, respectively. Similarly, the strength of the M1 and M3 samples was lower than that of the M2 and M4 samples. TEM bright field images of samples int eh T73 state displayed numerous coarsening intra-grain precipitates with ~9 nm in diameter. The η′ strengthening phase grows to a critical size, followed by transformation to the η phase with the composition of MgZn_2_, thereby resulting in a decline in strength. Additionally, the coarse precipitated phase loses coherent interaction with the matrix. It is easy to form a stress concentration area at the interface between the precipitated phase and the matrix [33,34,35,36]. It resulted in the elongation decreasing during tensile deformation. Thus, the elongation also decreased compare with the T6 state.

Diverse heating and cooling rates during solution treatment were observed to result in intermittent precipitation and wide PFZ along grain boundaries. When the sample was cooled to RT under different cooling rates, the degree of supersaturation of solute atoms in alloy matrix changed. The supersaturation of samples via water quenching (M2 and M4 samples) was higher than that of with air cooling (M1 and M3 samples). However, it did not affect the diffusion of solute atoms in local regions of grain boundaries. Under over-aging conditions, the sufficient atoms were driven by external driving forces [36]. These atoms segregated, resulting in the phase at the grain boundary becoming interrupted. Concurrently, the large widths of PFZ were formed in the grain boundaries.

The different heating and cooling rates also influenced the precipitation in the grain and the phase on the grain boundary during the subsequent natural aging state. The soluble phase redissolves in the matrix during the solution treatment. With air cooling (M1 and M3 samples), the alloy has a long residence time at high temperatures. Under the condition of sufficient diffusion driving force, the atoms of supersaturated solute are isolated and grow [37]. Coarse precipitates were formed in the grain and at the grain boundaries, as shown in Figure 8a,c and Figure 9a. In the following natural aging process, a large number of clusters did not form in the matrix. This was because the formation of coarse precipitates consumes super saturated solid solution atoms. Thereby, there were no more sufficient solute atoms to form clusters during natural aging. While, by using the water quenching method (M2 and M4 samples), the alloy remains in a state of high supersaturation. This resulted in a sufficient number of solute atoms diffusing at short distances during the natural aging process [38]. This results in the formation of high-density nanoscale clusters in the alloy matrix, as shown in Figure 8e. Notably, continuous precipitation on the grain boundary with the condition of high supercooling, as shown in Figure 9b.

## 5. Conclusions

In this study, the effects of different heating and cooling rates during solution treatment on the microstructure and properties of AA7050 alloy wires are revealed in detail. The results will help to optimize the solution treatment process of high-quality AA7050 alloy wires. The main conclusions can be summarized as follows:

(1) The size and uniformity of recrystallization microstructure were greatly affected by the heating rate during the solution treatment. The recrystallized grain of the samples with rapid heating was smaller and more homogeneous than that of slow heating.
(2) When the sample was cooled to RT using the air cooling method, the low density of the η′ strengthening phases formed after artificial aging treatment. While, using the water quenching method, sufficient solute atoms and numerous nucleation sites were provided, resulting in a large number of η′ strengthening phases being formed.
(3) The different heating and cooling methods affected the precipitation in the grain. And, the strength of the alloy wires is dominated by the precipitates. For the alloy in the T6 state, the tensile strength of alloy under the air cooling method reached ~375 MPa, and with the water quenching method it was ~585 MPa. For the alloy in the T73 state, the tensile strength of the former solution method was only ~310 MPa, while the strength of the latter increased to ~485 MPa.
(4) The intermittent precipitation and large widths of PFZ were detected along the grain boundaries. Coarse precipitates formed in the grain and at the grain boundaries under the air-cooling method. Less clusters were observed in the matrix during the following natural aging process, while high density nanoscale clusters and no continuous precipitation are formed when using the water quenching method.

## Figures and Tables

**Table 1:** The heat treatment methods of the samples with different heating and cooling rates.

Wire Samples	Solution Treatment	Aging Conditions
M1	Slow heating + Air cooling	T6 state
M2	Slow heating + Water quenching	
M3	Rapid heating + Air cooling	T73 state
M4	Rapid heating + Water quenching	

## Data Availability

The processed data required to reproduce these findings cannot be shared at this time, as the data also comprise a part of an ongoing study.
